# Psychometric properties of the vaccination fear scale (VFS-6) in Polish and their relationship with psychological distress

**DOI:** 10.3389/fpsyt.2025.1708085

**Published:** 2025-12-15

**Authors:** Olga Malas, Maciej Załuski, Marta Makara-Studzińska

**Affiliations:** 1 Department of Psychology, Sociology and Social Work, University of Lleida, Lleida, Spain; 2 Department of Bioethics and Health Psychology, Faculty of Health Sciences, Jagiellonian University Medical College, Kraków, Poland; 3 Department Health Science, Institute of Scientific Development, Vincent Pol University in Lublin, Lublin, Poland

**Keywords:** vaccination fear scale, VFS-6, distress, anxiety, depression, stress, vaccines

## Abstract

**Introduction:**

Preventing vaccine hesitancy is an important effort by the clinician. The present study aimed to evaluate the factorial structure and psychometric properties of the Vaccination Fear Scale (VFS) in a Polish sample, as well as to examine its measurement invariance across sex and its associations with psychological distress.

**Methods:**

A sample of 455 adults from the general population, broadly representative of the general Polish adult population, (Mean age = 37.05, SD = 13.14; 72.3% female) was recruited. Participants completed the seven-item version of the VFS. A subsample of 153 individuals also completed the 21-items Depression, Anxiety, and Stress Scale (DASS-21). Confirmatory factor analyses (CFA) were conducted to compare unidimensional, two correlated factors, and second-order two-factor models for 7-items and 6-items versions.

**Results:**

Results indicated that the 6-item version of the VFS (VFS-6), excluding item 5, provided superior fit compared to the 7-item version. The best-fitting model was a two-factor correlated structure (Cognitive factor: items 1, 2, and 4; Somatic factor: items 3, 6, and 7), confirming a clear cognitive-somatic structure. Measurement invariance analyses supported strict invariance between sexes. Internal consistency was high for both the total scale and its subfactors, and convergent and discriminant validity were satisfactory.

**Conclusion:**

Correlations with DASS-21 scores were low and non-significant, suggesting that vaccination fear is largely independent of general psychological distress measured as depression, anxiety and stress. These findings demonstrate that the VFS-6 is a reliable and valid instrument for assessing fear of vaccination in the Polish population. The results also clarify the role of item 5, and provide evidence for strict sex invariance, offering a robust tool for research and intervention planning.

## 1 Introduction

Fear, understood as a psychological construct, has been widely described as a fundamental modulating factor in human behavior. In health-related contexts it functions as a strong predictor of avoidance, rejection, or postponement of medical interventions (1). Previous research identifying fear as a crucial factor in vaccine hesitancy (2–4).

Fear of vaccination is a more consistent predictor of vaccination intention (3, 5, 6) than vaccination hesitancy (5) or fear of the disease (3), also showing a clear correlation with conspiracy beliefs (7). This fear is associated with a range of cognitive and emotional factors, from the perception of side effects to misconceptions about the risks and benefits of vaccines (8). These symptoms of fear are relatively easy to recognize and control, which makes it essential to measure both the emotional and physiological fear of vaccination as an indicator within strategies to increase vaccination rates worldwide (9). Considering these contextual factors, the present study focuses on the adaptation and validation of the Vaccination Fear Scale (3) for a Polish sample. Due to its compact structure, multidimensional approach, and robust validity and reliability, this tool has been widely used. However, to date, it has not been specifically validated in the Polish population.

The Fear of COVID-19 Scale (FoCV-19S), developed by Ahorsu et al. (10), was one of the first brief and valid tools to measure the construct of fear, in this case related to coronavirus. Due to its strong psychometric properties, this scale became the methodological basis for the development of instruments designed to assess fear of vaccination, the most significant being the Vaccination Fear Scale (VFS-6; 3) and the Fear of Coronavirus Vaccination Scale (FoCVVS; 9). Malas and Tolsá (3) adapted the structure of the Fear of COVID-19 Scale (FoCV-19S) to create the Vaccination Fear Scale (VFS-6), generalizing the items so as to measure fear of vaccination with any vaccine, including, but not limited to, the COVID-19 vaccine, or vaccination in general. During its validation in the Spanish population, one of the original items (item 5 of the FoCV-19S) was removed, as it showed inadequate factor loadings and ambiguity in its role within the structure, thus allowing for a more precise and consistent scale. Subsequently, the VFS-6 was adapted and validated in Italian (5), Arabic, English, Turkish, Ukrainian (11) and Bangla (12). Meanwhile, Ochnik et al. (9) developed the Fear of Coronavirus Vaccination Scale (FoCVVS) by complementing the items of the FoCV-19S to specifically assess fear of the COVID-19 vaccine, shifting the focus of fear from “COVID-19 in general” to “COVID-19 vaccination”. The FoCVVS was validated in several linguistic contexts, including German, Polish, Slovenian and Hebrew. Unlike the VFS-6, the FoCVVS retained item 5, which represents a key difference between the two scales.

During the validation of both the VFS-6 and the FoCVVS, exploratory factor analysis (EFA) was conducted. In the case of the VFS-6, Malas and Tolsá (3) obtained a two-correlated factors solution, with item 5 loading on both factors, which was the reason for its removal. As a result, a six-item scale with good internal structure and robust psychometric properties was obtained. Subsequent studies in other populations validated the 6-item factor (5, 11, 12), omitting the item removed by Malas and Tolsá (3) from their tests. By contrast, Ochnik et al. (9) found both an initial unifactorial solution and an alternative bifactorial solution, with item 5 loading on the somatic factor in the Polish sample, but on the cognitive factor in the German sample.

There was no consensus regarding the factorial structure of the original FoCV-19S, with some authors suggesting a unidimensional structure (13) and others a bifactorial one (14). For this reason, both studies tested all these factorial structures. As a result, Malas and Tolsá (3) validated the VFS-6 as a six-item scale with two correlated factors (Cognitive Factor: items 1, 2 and 4; Somatic Factor: items 3, 6 and 7), whereas Ochnik et al. (9) validated the FoCVVS as a seven-item bifactorial scale with two factors (Cognitive Factor: items 1, 2 and 4; Somatic Factor: items 3, 5, 6 and 7), this structure proving to be the best fit across all samples. Item 5 was not removed, despite its inconsistency in the German sample.

According to Malas et al. (11), the divergence regarding item 5 between the two scales may be due to the greater degree of adaptation carried out in the VFS-6 compared with the FoCVVS. This higher level of modification could explain why item 5 had to be removed in the VFS-6. However, this hypothesis has not yet been empirically tested, as analyses with the original seven items that gave rise to the VFS-6 have not been conducted in other languages. It is noteworthy that the FoCVVS was validated in Polish without the removal of item 5. Therefore, future studies should examine all seven items of the VFS to determine whether the removal of item 5 is indeed justified, addressing a current gap in the literature.

Once the version of the VFS (either with seven items or six items) with the best psychometric properties has been established, further studies on sex invariance will be necessary, since the existing evidence is scarce and inconclusive. For the VFS-6, Malas et al. (11) reported strict invariance between sexes in a multicultural sample of university students. However, in the general population, Duradoni et al. (5) found configural and metric invariance, while Hasanuzzaman et al. (12) only reported configural invariance.

Furthermore, although some studies have found low but significant correlations between fear of vaccination and generalized anxiety (11) or distress as anxiety, depression, and stress (12), these associations are weak and do not allow firm conclusions to be drawn regarding causal relationships or the underlying mechanisms. Moreover, recent research suggests that the effect of generalized anxiety on somatic and cognitive fear of vaccination may be mediated by other constructs, such as alexithymia, negative affect, or social inhibition (6). This highlights a gap in the research. It remains unclear whether anxiety or psychological distress directly affect the emotional response to vaccination. Understanding these potential relationships would be crucial for designing effective intervention and communication strategies in vaccination campaigns.

In this context, the primary aim of this study is to evaluate the seven original items of the Vaccination Fear Scale (VFS) in the Polish sample, in order to determine whether the most appropriate factorial structure of the scale should include six or seven items. Once the optimal version of the scale has been established, its invariance according to sex will be evaluated, given that the current evidence is limited and inconsistent. Furthermore, the relationship between fear of vaccination and psychological distress, measured in terms of depression, anxiety, and stress, will be explored, with the aim of determining whether these factors influence the emotional response to vaccination. The findings from this study will provide a validated tool for assessing fear of vaccination in Polish population and will also contribute to the broader literature on this construct and its psychological correlates.

## 2 Methods

### 2.1 Participants

Two datasets were collected from the general population. In the first dataset, 302 individuals participated (M = 35.56 years, SD = 11.97; 72.5% female). In the second dataset, 153 individuals participated (M = 40.00 years, SD = 14.79; 71.9% female). The combined sample comprised 455 individuals, aged 18 to 81 years (M = 37.05 years, SD = 13.14; 72.3% female, 27.7% male). The number of participants was deemed sufficient in light of established guidelines: Nunnally and Bernstein (15) recommend at least ten respondents per variable, while Tabachnick and Fidell (16) propose a minimum of 300 cases to ensure robust factor-analytic outcomes.

### 2.2 Procedure

In the first phase, the seven items originally proposed by Malas and Tolsá (3) for the VFS were translated into Polish. The translation and back-translation procedure for cross-cultural adaptation was carried out in accordance with the recommendations of the International Society for Quality of Life Assessment (IQOLA) (17). The translation and back-translation procedure is the first step in the three-step process alongside verification of the scaling requirements and validation of and establishing normative values for the adopted version of the questionnaire. The article describes the results of a validation study—an examination of a version of the measurement tool created during translation—leading to an assessment of the validity and reliability of the created tool.

The second phase involved data collection, which was conducted from March 21, 2024 to April 30, 2025 using a non-probabilistic convenience sampling strategy. The study procedure was a hybrid one. Both online and paper versions of the questionnaires were used. The online version of the survey was disseminated via IT platform of the Scientific Research System of the Jagiellonian University Medical College to reach a broad and heterogeneous group of respondents. The paper version of the survey was disseminated in one medical facilities. Eligible participants were required to be adults and fluent in Polish, with no additional inclusion or exclusion criteria. Two datasets were collected: in the first (n=302) participants completed the VFS; in the second (n = 153), participants completed both the VFS and the DASS-21. The first dataset was obtained online, the second using the paper version of the questionnaires.

Prior to completing the online questionnaire, participants were provided with an information about the study’s objectives, procedure, and assurances of confidentiality. Information was provided both online and paper format. Informed consent was obtained from all participants, who voluntarily agreed to take part in the research. Those who did not provide consent were denied access to the study. Ethical approval was secured from the Research Ethics Committee of the Jagiellonian University Medical College (Approval No 118.0043.1.59.2024 from March 21, 2024).

### 2.3 Instruments

#### 2.3.1 Vaccination fear scale

The VFS-6 is a six-item scale developed by Malas and Tolsá (3) to assess fear of vaccination in the general Spanish population. The scale was developed from seven items, but item 5 was removed following the exploratory factor analysis. In the present study, the seven-item was administered to examine whether the six-item version truly offers psychometric advantages over the seven-item version. The scale can be adapted to measure general fear of vaccination or tailored to a specific vaccine by inserting its name. In this study, it was adapted to assess fear of the COVID-19 vaccine Pfizer-BioNTech mRNA (Comirnaty).

The VFS is a two correlated factors tool: cognitive factor including question on cognitive symptoms (F1: items 1, 2, and 4) and somatic factor including question on somatic symptoms (F2: items 3, 5, 6, and 7). Items are rated on a 5-point Likert scale, from 1 (strongly disagree) to 5 (strongly agree), with total scores ranging from 6 to 30 for the six-item version and from 7 to 35 for the seven-item version. Higher scores indicate greater levels of fear related to vaccination. Cronbach’s α coefficients reported in previous studies for the total scale range from 0.81 to 0.91 (3, 5, 11, 12). Subscale reliabilities have typically remained within this range, although slightly lower coefficients were obtained in the Bangla sample, with α = 0.75 for the Cognitive Factor and α = 0.80 for the Somatic Factor (12). In this study, internal consistency was assessed for both the total scale and its subscales in the seven-item and six-item versions, with Cronbach’s α exceeding.840 in all cases (see **Table 1**).

**Table 1:** Fit indices (IC 90%), for different models of VFS with 7-items and 6-items and invariance analysis for sex, reliability and validity in the best identified model (n = 455).

	Model 1	Model 2	Model 3	Model 4		Model 5	Model 6	Model 7	Model 8
AIC	9216	9053	9060	7866		7716	7718	7693	7685
BIC	9303	9147	9155	7940		7795	7810	7775	7771
*χ²/df* *(p)*	20.45 (<.001)	9.90 (<.001)	10.53 (<.001)	22.67 (<.001)		6.60 (<.001)	7.54 (<.001)	3.91 (<.001)	2.91 (.008)
CFI	0.871	0.949	0.946	0.887		0.974	0.974	0.988	0.993
TLI	0.807	0.912	0.905	0.812		0.951	0.943	0.975	0.983
RMSEA	0.207	0.140	0.145	0.218		0.111	0.120	0.080	0.065
RMSEA IC90%	0.180/.23	0.12/0.16	0.12/0.17	0.19/0.24		0.08/0.14	0.09/0.15	0.05/0.11	0.03/0.10
SRMR	0.053	0.046	0.049	0.050		0.033	0.033	0.027	0.015
Model 8 (=VFS-6): sex invariance									
	CFI		ΔCFI			RMSA		ΔRMSEA	
Configural	0.992		–			0.070		–	
Metric	0.990		-0.002			0.066		-0.004	
Scalar	0.987		-0.003			0.068		0.002	
Strict	0.987		0.000			0.059		-0.009	
Model 8 (= VFS-6) reliability and validity data									
	F1		F2		VFS-6				
Coefficient α	0.849		0.863		0.900				
Coefficient ω	0.801		0.810		0.882				
AVE	0.68		0.64		–				
HTMT	0.849				—				

Model 1: Unifactorial model for VFS-7. Model 2: Two correlated factors for VFS-7 (F1: items 1, 2 & 4; F2: items 3, 5, 6 & 7). Model 3: Two-factor second-order model for VFS-7. Model 4: Unifactorial model for VFS-6. Model 5: Two correlated factors for VFS-6 (F1: items 1, 2 & 4; F2: items 3, 6 & 7). Model 6: Two-factor second-order model for VFS-6. Model 7: Two correlated factors for VFS-6 (F1: items 1, 2 & 4; F2: items 3, 6 & 7) with correlated errors for items 6 & 7. Model 8: Two correlated factors for VFS-6 (F1: items 1, 2 & 4; F2: items 3, 6 & 7) with correlated errors for items 6 & 7, and items 1 & 2. CFI= Comparative Fit Index; TLI= Tucker-Lewis Index; RMSEA= Root mean square error of approximation; SRMR= Standardized root mean square residual. F1 = Cognitive Factor for VFS-6. F2 = Somatic Factor for VFS-6. VFS-6 = 6-items Vaccination Fear Scale. DASS = 21-items Depression, Anxiety and Stress Scale. AN = Anxiety measured with DASS-21. DE = Depression measured with DASS-21. ST, Stress measured with DASS-21; DAS, Distress measured with DASS-21 (AN+DE+ST).

#### 2.3.2 Depression, anxiety and stress scale

This is a self-report questionnaire originally validated by Lovibond and Lovibond (18) that assesses emotional distress across three seven-item subscales: depression, anxiety, and stress. Each of the 21 items is rated on a four-point Likert scale, reflecting both the intensity and frequency of emotional distress experienced over the past week (0 = did not apply to me at all; 3 = applied to me very much, or most of the time). Higher scores indicate greater severity or frequency of distress. Each subscale yields a separate score ranging from 0 to 21, with scores above 14 for depression, 10 for anxiety, and 17 for stress indicating extremely severe levels. The Polish version of the scale was used in the study (19). Cronbach’s α coefficients the polish version of the scale was 0.93 for the total DASS-21 and 0.86, 0.84, and 0.85 for the DASS-21 Depression subscale, DASS-21 Anxiety subscale, and DASS-21 stress subscale, respectively (19). In the present study, the scale exhibited Cronbach’s α of 0.881 for anxiety, 0.920 for depression, 0.896 for stress, and 0.956 for the total scale.

### 2.4 Statistical analysis

All analyses were conducted using JASP software (version 0.95.1.0) with the Lavaan add-on.

In the first phase, the data obtained for the VFS from the two collected databases were used (n = 455).and the factorial structure was examined using confirmatory factor analysis (CFA) with the maximum likelihood (ML) estimation method. Taking into account the models described for Malas and Tolsá (3) and Ochnik et al. (9), three models were tested: a unidimensional model, the two-correlated factor model (Cognitive factor; items 1, 2 and 4; and Somatic factor: items 3, 5, 6 and 7 for VFS-7 or items 3, 6 and 7 for VFS-6) and their second-order two-factor model. Model fit was evaluated using the χ²/df, although it is sensitive to sample size, Tucker–Lewis Index (TLI), Comparative Fit Index (CFI), Root Mean Square Error of Approximation (RMSEA), and Standardized Root Mean Square Residual (SRMR). Following Hu and Bentler (20), χ²/df values below 3 was recommended, TLI and CFI values above 0.95 and RMSEA values below 0.06 indicate good fit, and values of TLI and CFI above 0.90 and RMSEA values below 0.08 are considered acceptable. For SRMR, values below 0,08 is considered acceptable. AIC and BIC were also determined and used to compare nested models.

In a second phase, measurement invariance (configural, metric, scalar, and strict) was conducted across sexes on the model that showed the best fit indices. It was examined using multi-group confirmatory factor analysis (MGCFA). Factorial invariance between groups was assessed by comparing the equality of parameters in the measurement model, including factor structure, factor loadings, intercepts, and residuals (21). Starting from the baseline model, invariance constraints were applied sequentially. Configural invariance ensures that the factor structure and pattern of factors are equivalent across all groups. Metric, scalar, and strict invariance were tested by progressively constraining factor loadings, item intercepts, and measurement errors, respectively. Constrained models were compared with the unconstrained model using fit indices, with invariance considered supported if ΔCFI ≤ 0.01 and ΔRMSEA ≤ 0.015 (22).

Following, construct validity of the scale was evaluated by assessing reliability using both Cronbach’s α and McDonald’s ω for the total scale and its subscales, with α > 0.80 and ω > 0.75 considered acceptable (23). Convergent validity was examined through the average variance extracted (AVE), with values of 0.50 or higher indicating satisfactory convergence (23). Divergent validity was assessed by analyzing the heterotrait–monotrait (HTMT) ratio, with values below 0.85 considered indicative of adequate discriminant validity (24).

Finally, descriptive statistics, including means, standard deviations, skewness, and kurtosis, were calculated for the individual items of the VFS (n= 455). For the scales and subscales of the VFS and DASS-21 the descriptive analysis was carried out using data from the second database (n = 153), as it was the only one that included DASS-21 data. For scales and subscales also the Shapiro–Wilk test was conducted to evaluate normality. Following, for the best-fitting model, Spearman’s rho correlations were applied for two purposes. The first was to provide evidence of construct validity, showing that the factors of the VFS adequately represent the cognitive and somatic dimensions of fear of vaccination. The second was to establish concurrent validity with the DASS-21 and its subscales (anxiety, depression, and stress), investigating potential associations between psychological distress and fear of vaccination.

## 3 Results

### 3.1 Confirmatory factor analysis

Fit indices for all tested models are presented in **Table 1**. As can see, the unifactorial model of the VFS-7 (Model 1) and the VFS-6 (Model 4) showed insufficient fit in both cases (CFI/TLI <.90; RMSEA > 0.10), indicating that a single factor does not adequately explain the structure of the items.

The two correlated factor models of the VFS-7 (Model 2) and the VFS-6 (Model 5) considerably improved the fit compared to the unifactorial models (CFI/TLI >.90; RMSEA > 0.10). In turn, the second-order two-factor models of the VFS-7 (Model 3) and the VFS-6 (Model 6) showed similar fit to the correlated two-factor models, suggesting that a general factor may explain the covariation between first-order factors, although without substantially improving the fit compared to the correlated model. Overall, the VFS-6 models (Models 4, 5, and 6) presented superior fit to their VFS-7 counterparts (Models 1, 2, and 3), suggesting that the reduced version more clearly represents the factorial structure. Among the VFS-6 models, Model 5 (two correlated factors) showed the best initial fit and was therefore selected as the basis for further improvement by correlating specific item errors. Models 7 and 8, which includes additional error correlations, achieved the best overall fit (CFI/TLI >.95; RMSEA = .080 and.065 respectively), indicating that some items share residual variance not explained by the factors.

**Figure 1:**
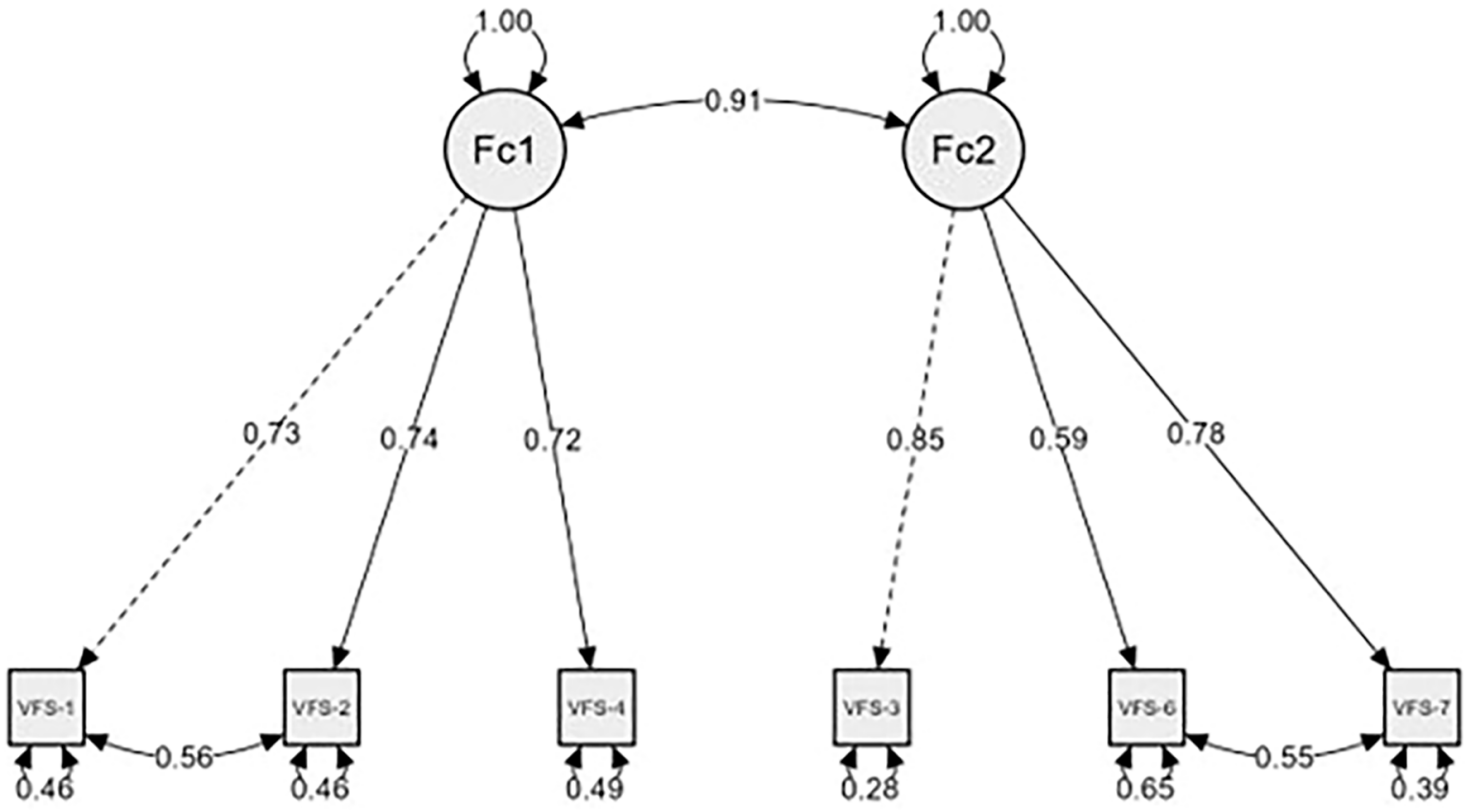
Factor structure of the VFS-6 scale (Model 8). Note that: Fc1= Cognitive factor. Fc2= Somatic factor (n = 455).

Although the interpretation of model fit focuses primarily on CFI, TLI, and RMSEA, other indices reported in **Table 2** provide complementary information. The χ²/df statistic, while sensitive to sample size, indicates reasonable to good fit for Models 7 (3.91) and 8 (2.91), supporting the adequacy of the two correlated factor models with correlated errors. SRMR values are within acceptable limits, further confirming model adequacy. AIC and BIC are particularly relevant for comparing nested models, such as the VFS-6 relative to the VFS-7, and show that the reduced scale achieves better parsimony while maintaining good fit. Overall, these additional indices corroborate the conclusions drawn from CFI, TLI, and RMSEA, confirming that Model 8 provides the best overall representation of the cognitive-somatic structure of the VFS-6 (see **Figure 1**).

**Table 2:** Descriptive Statistics and Internal Consistency of VFS-6 items (n = 455) and Descriptive Statistics and Spearman Correlations for VFS-6 and DASS-21 scales and their subscales (n = 153).

VFS Items	Mean		SD	Skewness	Kurtosis	Corr.		Coef. α	
1	2.218		1.404	0.730	-0.894	0.721		0.850	
2	2.314		1.427	0.696	-0.919	0.689		0.856	
3	1.796		1.233	1.358	0.516	0.708		0.854	
4	2.892		1.420	0.098	-1.308	0.650		0.863	
6	1.714		1.305	1.659	1.262	0.614		0.867	
7	1.844		1.312	1.312	0.269	0.732		0.848	
Scales/subscales	Mean		SD	Skewness	Kurtosis	S-W (p-value)			
F1	7.424		3.727	0.487	-1.004	0.909 (<.001)			
F2-	5.354		3.412	1.289	0.359	0.724 (<.001)			
VFS-6	12.778		6.626	0.878	-0.364	0.873 (<.001)			
AN	1.853		3.928	2.480	5.780	0.548 (<.001)			
DE	2.020		4.223	2.427	5.298	0.552 (<.001)			
ST	2.800		4.962	1.783	2.195	0.633 (<.001)			
DAS	6.673		12.570	2.104	3.777	0.607 (<.001)			
Spearman’s rho (p-value)									
Variable	F1	F2		VFS-6	AN	DE	ST		DAS
F1	–								
F2	0.681 (<.001)	–							
VFS-6	0.947 (<.001)	0.851 (<.001)		–					
AN	0.069 (.393)	0.031 (.700)		0.051 (.535)	–				
DE	0.021 (0.800)	-0.010 (0.903)		-0.005 (0.955)	0.739 (<.001)	–			
ST	0.031 (0.706)	0.010 (0.898)		0.036 (0.663)	0.728 (<.001)	0.765 (<.001)			
DAS	0.027 (0.739)	0.015 (0.857)		0.032 (0.694)	0.886 (<.001)	0.911 (<.001)	0.923 (<.001)		–

SD, Standard Deviation. Corr, Corrected item total correlation. Coef. α, Coefficient α if item dropped. S-W, Shapiro-Wilk. F1 , Cognitive Factor for VFS-6. F2 , Somatic Factor for VFS-6. VFS-6 , 6-items Vaccination Fear Scale. DASS , 21-items Depression, Anxiety and Stress Scale. AN , Anxiety measured with DASS-21. DE, Depression measured with DASS-21. ST: Stress measured with DASS-21. DAS, Distress measured with DASS-21 (AN+DE+ST).

### 3.2 Invariance analysis

Measurement invariance across sex was evaluated using Model 8 (VFS-6, with correlated error pairs 1–2 and 6–7), as it showed the best fit indices. Factorial invariance was assessed through configural, metric, scalar, and strict models. As shown in **Table 1**, the fit indices for each model were adequate and the changes between them met the established specifications (ΔCFI ≤.010 and ΔRMSEA ≤.015). These results indicate that the VFS-6 achieves configural, metric, scalar, and strict invariance between men and women, supporting the comparability of latent means and variances of the instrument across groups.

### 3.3 Reliability and validity data

As shown in **Table 1** the VFS-6 demonstrated good psychometric properties. Internal consistency was high, with both Cronbach’s α and McDonald’s ω exceeding 0.80 for the total score and its subfactors, indicating that the items reliably measure the intended constructs. Convergent validity was satisfactory, as reflected by AVE values above 0.50 for all factors, while discriminant validity was supported by HTMT ratios below 0.85, confirming that the subfactors capture distinct aspects of vaccination fear.

### 3.4 Descriptive statistics

**Table 2** presents the descriptive statistics of the Vaccination Fear Scale (VFS-6) items, its total scores and subscales, as well as the Depression, Anxiety and Stress Scale-21 (DASS-21) and its subscales.

The means of the individual VFS items ranged from 1.71 to 2.89, with standard deviations between 1.23 and 1.49, indicating that responses tended to cluster at the lower end of the scale, although with moderate variability. Item 4, belonging to the cognitive factor, showed the highest mean (2.89), while items 3, 6, and 7, from the somatic factor, were the lowest (1.7–1.8). Taken together, these results suggest that participants reported generally low levels of vaccination fear, but with relevant individual differences.

The VFS subscales showed low to moderate means, and the total scores were also in the moderate range. In all cases, standard deviations were relatively large in relation to the mean, indicating heterogeneity in the responses. Overall, the data reflect a generally low perception of vaccination fear, but with sufficient variability for analysis.

The means of the DASS-21 subscales were also low. The total score likewise remained low on average, but with very high standard deviations. This indicates that while most participants reported low levels of psychological distress, there were isolated cases with higher scores that increased dispersion. These results are consistent with what is typically observed in general non-clinical populations, where the prevalence of symptoms of anxiety, depression, and stress is usually low, although individuals with elevated scores may still be present.

In the items of the Vaccination Fear Scale (VFS), the somatic items (3, 5 and 6) exhibit strong positive skewness and high kurtosis, indicating a concentration of low scores with pronounced peaks, whereas the cognitive items (1, 2, and 4) show moderate positive skewness and flatter distributions. In the VFS subscales and total scores, positive skewness is moderate and kurtosis is mild; in all cases, Shapiro-Wilk tests (p <.001) confirm non-normal distributions. Similarly, in the DASS-21, all subscales and the total score show very strong positive skewness and high kurtosis, reflecting that most participants report low levels, with some extreme cases. Shapiro-Wilk tests confirm the absence of normality, a pattern expected in general non-clinical populations. It can be concluded that the items, subscales, and total scores of both the VFS and DASS-21 exhibit non-normal distributions. Consequently, the correlation analysis between the VFS-6 scale and subscales and the DASS-21 scale and subscales was conducted using Spearman’s rho.

Internal consistence date for the VFS-6 presented Cronbach’s α values if item dropped between 0.848 and 0.867. Corrected item-total correlations ranged from 0.614 to 0.732. In conclusion, the VFS-6 demonstrate high reliability and are suitable for assessing vaccination fear in general population samples.

### 3.5 Spearman’s correlations

Construct validity was further evidenced by significant Spearman’s rho correlations between the total score and the subfactors (see **Table 2**), demonstrating that the factor structure accurately represents the underlying cognitive and somatic dimensions. In contrast, concurrent validity with the DASS-21 and its subscales for anxiety, depression, and stress was low and non-significant, suggesting that vaccination fear, as measured by the VFS-6, is largely independent of general psychological distress in this sample. Overall, these findings indicate that the VFS-6 is a reliable and valid instrument for assessing vaccination fear, with a clear cognitive-somatic structure.

## 4 Discussion

The objectives proposed in this study were successfully achieved. The primary aim was to evaluate the factorial structure of the VFS in a Polish sample to determine whether a six- or seven-item version best represents the construct. Confirmatory factor analyses indicated that the six-item version (VFS-6) provided superior fit compared to the seven-item version. In this way, the findings of the present study align closely with previous research on the VFS-6 and related instruments. Consistent with Malas and Tolsá (3), the factorial analyses confirmed a two-factor correlated structure, separating cognitive and somatic components of vaccination fear. The removal of item 5, as in the original Spanish validation, also improved model fit, supporting the idea that this item may contribute ambiguity within the scale’s structure. These results replicate and extend findings from other linguistic adaptations, including Italian, Arabic, English, Turkish, Ukrainian, and Bangla populations (5, 11, 12), reinforcing the robustness and cross-cultural applicability of the six-item version. While the FoCVVS, validated by Ochnik et al. (9), showed a second-order two-factor solution in the Polish sample, with item 5 loading on the somatic factor (and on the cognitive factor in the German sample), the current adaptation of the VFS-6 confirms that excluding item 5 results in a clearer cognitive-somatic structure. This difference may support the hypothesis proposed by Malas et al. (11), according to which the greater degree of adaptation applied by Malas and Tolsá (3) to the original items could explain the observed divergence with the FoCVVS and item 5.

Regarding measurement invariance, the present study extends previous findings by demonstrating strict invariance across sex for the VFS-6 in a general Polish population. Prior studies reported partial evidence of invariance: Malas et al. (11) found strict invariance in a multicultural student sample, while Duradoni et al. (5) observed only configural and metric invariance, and Hasanuzzaman et al. (12) reported configural invariance. The current results therefore provide robust support for comparing vaccination fear across sexes in general populations, addressing a gap identified in earlier research.

Contrary to expectations, vaccination fear was not significantly correlated with psychological distress, measured as depression, anxiety, or stress. This finding differs from previous studies, which reported weak but significant positive correlations between these constructs and vaccination fear (11, 12). These results suggest that general anxiety or distress may not be reliable predictors of vaccination fear. Nevertheless, as highlighted by Malas et al. (6), these factors could still influence vaccination fear indirectly through other variables, such as alexithymia, negative affect, or social inhibition. Moreover, other determinants, such as needle phobia (25, 26) and conspiracy beliefs (7, 25), should also be considered when examining the emotional response to vaccination.

Despite its contributions, this study has several limitations that should be acknowledged. First, participants were recruited through non-probabilistic convenience sampling via only one IT platform and a group from one medical facility, which may introduce selection bias and limit the generalizability of the findings to the broader Polish population. Second, other limitation is the sex imbalance in the sample. Among the 455 participants, only 27.7% were male. In the same way the subsample used for analyses involving the DASS-21 was smaller (n = 153), potentially reducing statistical power for detecting associations with depression, anxiety, and stress. Third, all data were collected via self-report questionnaires, which may be subject to social desirability and reporting biases, particularly regarding sensitive topics such as vaccination fear. Fourth, the proportion of participants aged over 65 years was very low, limiting the generalizability of the findings to older adults. Fifth, psychological distress was measured solely with the DASS-21, without incorporating other potentially relevant constructs such as needle phobia or conspiracy beliefs, which may also contribute to vaccination fear.

Consequently, future research should aim to recruit larger and more representative samples, including older adults, to enhance generalizability. Longitudinal designs could help clarify causal relationships between vaccination fear and psychological or cognitive factors. Additionally, incorporating a broader range of psychological constructs, such as needle phobia, conspiracy beliefs, alexithymia, and negative affect, would provide a more comprehensive understanding of the determinants of vaccination fear and inform targeted intervention strategies.

Despite these limitations, the study confirms the psychometric robustness of the VFS-6, supports its cross-cultural adaptation, clarifies the role of item 5, and strengthens evidence for strict sex invariance. These findings contribute to a more precise measurement of vaccination fear and provide a validated tool for research and intervention planning in the Polish context.

## Data Availability

The raw data supporting the conclusions of this article will be made available by the authors, without undue reservation.
